# Quantifying and Predicting the Spread of Established Non-Native Fishes in Peninsular Florida, USA

**DOI:** 10.3390/biology14020189

**Published:** 2025-02-12

**Authors:** Katelyn M. Lawson, Hannah G. Talbert, Jeffrey E. Hill

**Affiliations:** 1 Program in Fisheries and Aquatic Sciences, Tropical Aquaculture Laboratory, School of Forest, Fisheries, and Geomatics Sciences, University of Florida, 1408 24th Street SE, Ruskin, FL 33570, USA; jeffhill@ufl.edu; 2 Department of Crop, Soil, and Environmental Sciences, 201 Funchess Hall, Auburn University, Auburn, AL 36849, USA; hgt0034@auburn.edu

**Keywords:** life history, invasive species, alien species, risk assessment

## Abstract

The spread of non-native species plays a substantial role in the designation of a species as invasive, yet the determination and measurement of non-native-species spread is challenging, particularly for fishes, which are limited by aquatic connectivity. Spread has been quantified for fishes in a variety of ways and exact methods vary by region and taxonomic group. In this study, we quantified fish spread in peninsular Florida and used life history traits to understand what factors contribute to the rate at which fish species spread. Using a variety of statistical analyses, we found that fast spreaders in peninsular Florida tend to have a larger body size, narrow diet, shorter time to hatch, greater salinity tolerance, and higher fecundity. However, some variables like parental care, egg diameter, and reproductive guild were the same or very similar across all established species that were included in the analyses. Predicting whether an established species will spread quickly or slowly in Florida may be more challenging than predicting whether an introduced fish species will establish, yet there is support across regions for the use of life history traits in the risk assessment process.

## Simple Summary

Non-native species become invasive after they spread beyond the point of introduction, and especially when they have social or environmental impacts. Understanding the characteristics of fish species that spread quickly versus slowly can help managers prioritize the efforts for Early Detection Rapid Response actions. First, we measured the extent of spread for each species by calculating the area of clustered occurrences. We then examined the traits of non-native freshwater fish species in Florida that have established and spread, splitting the list of established fish species into fast and slow spreading categories. We found that all but one of the 31 established species included in the analyses care for their young, a trait highly predictive of successful establishment but not useful for differentiating fast and slow spreaders. Instead, fast-spreading species tended to have a narrower diet and larger body size. Many of the fast-spreading species also have a tolerance for low salinity. Identifying these characteristics will help managers better understand the potential risks of newly introduced and established freshwater fish species in peninsular Florida.

## 1. Introduction

A non-native species must complete several stages of an invasion process before it can be designated as “invasive” [1]. The most widely used stages of the invasion process are introduction, reproduction, establishment, spread, and impacts, although additional stages such as transport, recruitment, dispersal, and integration can be found in the literature [2,3,4,5]. A species is not typically classified as invasive until it spreads and causes documented impacts. Rejmanik et al. [6] defines an invasive species as a non-native species that spreads beyond the introduction site and becomes abundant. Species that maintain localized populations at introduction sites are non-invasive, and those that become widespread are considered invasive [1]. This definition is widely accepted in academia; however, governmental agencies may use different legal and management definitions [7]. For example, the USA Executive Order 13112 defines an invasive species as one that is non-native to the ecosystem under consideration and whose introduction causes or is likely to cause economic or environmental harm or harm to human health [8]. This difference highlights the importance of examining each stage independently [3].

Life history traits have been used across taxa to predict the spread of non-native species [9]. No traits have consistently been predictive of the successful completion of invasion process stages across taxa and region; regional analyses for specific taxonomic groups are most effective [3,9,10]. Predictive traits for freshwater fishes are varied and include physiological tolerances [11,12], trophic status [13,14], and life span or body size [13,14]. The use of a variety of traits to analyze this stage of invasion independently is important because traits which contribute to successful establishment may not also contribute to spread [3].

Evaluating the spread of non-native species is crucial for understanding invasiveness but methods used for measuring spread have differed across taxa. For plants, invasiveness is primarily based on degree of spread [15]. In most studies that used traits to predict plant invasion, invasiveness (yes/no) was determined a priori based on whether the species had spread beyond the site of introduction into natural areas [15,16,17,18]. The spread of plants and exclusively terrestrial species across taxa may simply be measured as area (ha) of infestation or occurrence; e.g., [19]. For freshwater fishes, the process of spread and its quantification is more difficult and comparatively few studies have examined this stage [3]. Freshwater fishes have complex spread dynamics influenced by permanent and intermittent waterbody connectivity, often requiring overcoming physical obstacles such as waterfalls, dams, control structures, or land, or physiological barriers of salinity, temperature, or water chemistry [20,21]. Spread is frequently channeled due to the linearity of waterways and limited aquatic connectivity; therefore, it is difficult in many cases to adequately describe.

Quantifying and predicting the spread of non-native fishes has not been attempted in many studies, particularly when compared to other stages of the invasion process such as establishment [3]. The spread of non-native freshwater fishes has been generally modeled using spread as a continuous variable in multiple linear regression [12,13,14] or as a binary variable in multiple logistic regression [11]. Published methods for quantifying the spread of non-native fishes include calculating (1) the number of counties [22] or catchments [12,13,14] occupied by a species, (2) the number of lakes occupied by a species over time [11], (3) the rate of spread by a species in a river in kilometers per year [23], and (4) the river length occupied by a species [24]. Nearly all studies have focused on temperate or cold-climate regions where the climate, habitat, and invading fauna are different from that of Florida. These methods may not work for subtropical regions with little topographical variation and highly connected hydrology.

Florida (USA) is a hotspot for freshwater fish introductions with about 48 established species [25,26]. Florida’s combination of climate, topography, and hydrology is favorable for the establishment of non-native fish species [27,28,29]. These factors also complicate analysis of the spread. South Florida’s tropical climate is generally conducive for species from a wide range of climate types, with northward expansion limited by cold winter temperatures in central or northern Florida [30,31]. Florida has diverse water bodies including large rivers, streams, springs, lakes, wetlands, canals, and estuaries. The topography is relatively flat, with weak drainage divides between some basins. Wet and dry season dynamics drive seasonal hydrology, resulting in changing local and regional connectivity and surface area of water bodies. This diversity of water bodies presents some challenges for quantifying spread according to previously published methods. For example, Florida’s watersheds vary greatly in size which precludes their use as a measure of spread. When using the hydrologic unit code (HUC) 8 map, south Florida is divided into only three large watersheds: Big Cypress Swamp (7375 km^2^), Everglades (11,449 km^2^), and Florida Southeast Coast (8096 km^2^). In contrast, HUC 8 watersheds in the Tampa Bay area are much smaller: Manatee River Drainage (944 km^2^), Little Manatee River Drainage (587 km^2^), Alafia River Drainage (1091 km^2^), and Hillsborough River Drainage (1699 km^2^). Florida has more than 7500 lakes, 12,000 miles of rivers, streams, and canals, and now contains over 100,000 stormwater ponds [32,33]. This immense number of water bodies and their varying size and connectivity make the approach taken by Kolar and Lodge [11] not feasible for measuring the spread of non-native fishes in Florida. Given these difficulties, a novel approach to quantifying spread is needed.

The spread of non-native species plays a substantial role in the designation of a species as “invasive”. Therefore, quantifying spread as accurately as possible is important. Life history traits have been successfully used to predict the success and failure of non-native freshwater fish in Florida up to the spread stage [34,35]. We took the next logical step to attempt to model spread using a framework of life history traits. Understanding the risk of a non-native species’ spread helps inform Early Detection Rapid Response decisions and predicting the potential range in the invaded region. Methods used in other regions seem unlikely to be adequate for Florida, so we developed a new method of quantifying spread and used a life history framework for describing/modeling spread. Our objectives were to (1) develop methods to quantify the spread of established non-native fishes in Florida, and (2) predict spread of established non-native fish species using life history traits.

## 2. Materials and Methods

To examine the spread of established non-native freshwater fishes, we identified 31 of approximately 40 established species in peninsular Florida [26,29] with adequate data for inclusion in the analyses (Table 1). We included 15 predictor variables in the analyses and data were gathered from the primary literature when available, and databases and other sources when necessary [34,35] (Table 2). Due to small sample size, correlations among variables were not calculated and no variables were removed prior to predictive analyses.

**Figure 1:**
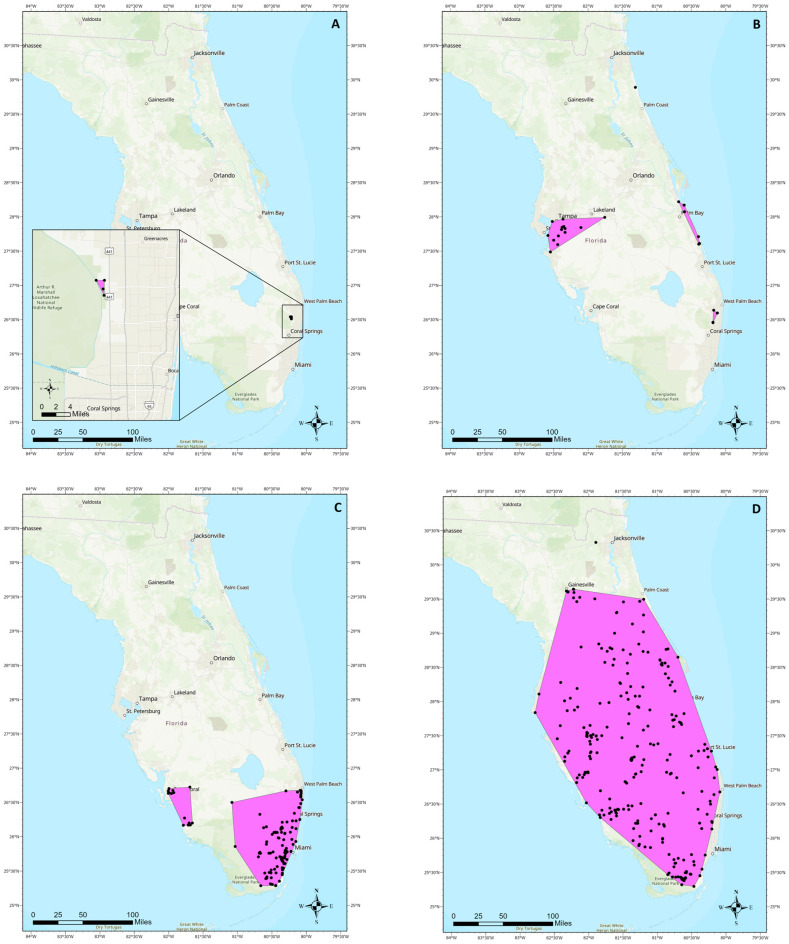
Maps with established species distribution examples and polygons used for calculating spread areas. (**A**) croaking gourami *Trichopsis vittata*, a slow-spreading, highly localized population. (**B**) green swordtail *Xiphophorus hellerii*, a series of slow spreading, localized, or extirpated populations across the state. (**C**) butterfly peacock bass *Cichla ocellaris*, a fast-spreading but thermally limited species. (**D**) brown hoplo *Hoplosternum littorale*, a species which has quickly spread throughout much of the state. Black points represent collection locations for each species.

We developed a new method for quantifying non-native freshwater fish species spread in Florida that could be used in other geographic regions in place of previous methods in the literature. First, collection data from the U.S Geological Survey (USGS) Nonindigenous Aquatic Species (NAS) database [36] were imported into ESRI ArcGIS Pro 3.0 (Redlands, CA, USA). Only the locations where status was listed as “established” were retained for analysis. Clusters with a minimum of three points were identified and any point more than 50 km away from its closest neighbor was excluded from the cluster. The Minimum Bounding Geometry tool was used to create a minimum convex polygon for each cluster (Figure 1). The area of each constructed polygon was calculated, and the total area of the polygons for a species was divided by the number of years since its first collection record in the state. The result of these calculations was the number of square kilometers a species has spread per year (Table 1).

**Figure 2:**
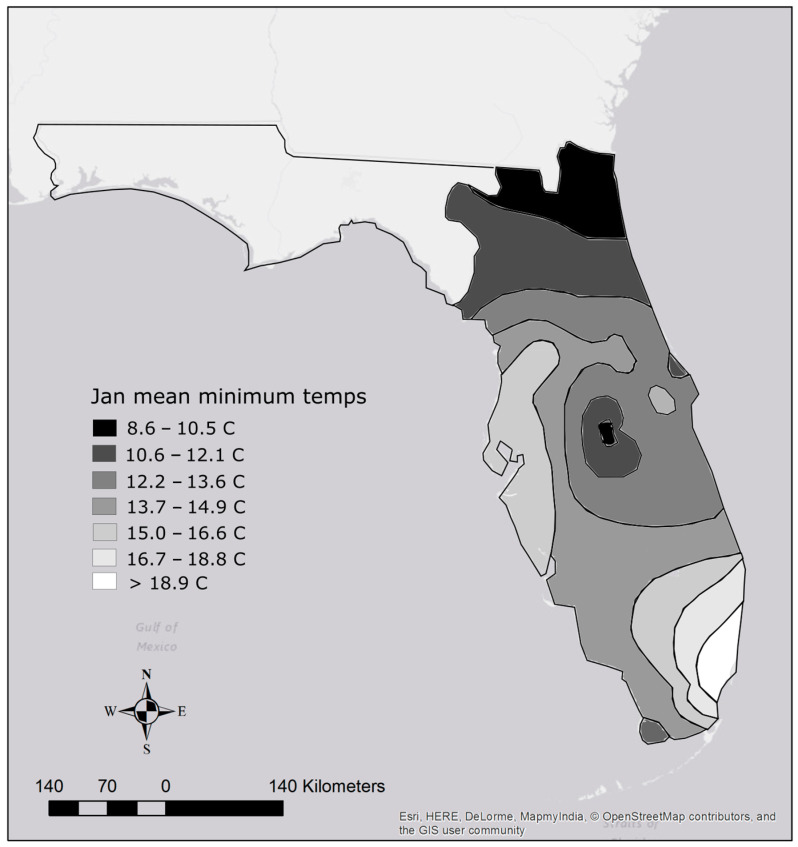
Map of Florida with temperature isolines for areas east of the Suwannee River Basin. Temperatures are based on the map in Lawson et al. [28].

Thermal tolerance affects the ability of an organism to spread. Therefore, using the previous methodology, some species appear to be slow spreaders, but only because they are thermally limited, and their potential range is small. From a state-wide risk perspective, this may not be important; however, from a biological perspective, this is an issue that requires attention. To address this deficiency, we first determined each species’ potential range based on their lower lethal temperature. A map was constructed by Lawson et al. [28] using January mean minimum water temperatures to interpolate the thermal minima of Florida’s regions. Polygons were constructed according to the temperature ranges presented in Lawson et al. [28] and the area of each polygon was calculated (Figure 2). For each species, the area of the polygons with a minimum temperature higher than its lower lethal were summed to obtain its potential range. We then took each species’ previously calculated area occupied and divided it by their potential range in peninsular Florida to obtain a percentage, then divided that percentage by the number of years since introduction. This resulted in the percent potential range occupied per year since introduction (Table 1).

We determined a cutoff value of 0.16 where anything at or above that value was classified as a fast spreader, and anything below was classified as a slow spreader (Table 1). That value is close to the one used by Kolar and Lodge [11] and fit the data most appropriately based on our observations and interpretation of spread. Logistic regression models were created using these classifications and the life history traits identified from the previous studies using the R package ‘caret’ [34,35,36]. A bi-directional stepwise selection method was then used to identify the best model using AIC values [37]. The Mann–Whitney U test was used to determine significant trait differences between species that are fast spreaders versus those that are slow spreaders [38]. The results of the Mann–Whitney U tests were used to construct additional logistic regression models based on significant differences. To visualize the contribution of different traits toward differentiating fast versus slow spreaders, we used canonical discriminant analysis, a multivariate method that determines the relationship between a categorical variable and independent variables of mixed data types (Table 2). Three variables, swim factor, length of spawning season, and air breathing, were removed from the analysis as they did not contribute to the differentiation of fast- versus slow-spreading species. Canonical scores and structure coefficients for the discriminant function were plotted using the R package ‘candisc’.

## 3. Results

Of the 31 established freshwater fish species examined, over half (n = 18) were classified as slow spreaders and nearly half (n = 13) were fast spreaders (Table 1). The first species with a record of establishment in Florida, 84 years ago, was the Texas cichlid *Herichthys cyanoguttatus* and the most recent was the Nile tilapia *Oreochromis niloticus* which was first documented as established 18 years ago (Table 1). The most widespread species included blue tilapia *Oreochromis aureus*, brown hoplo *Hoplosternum littorale*, and walking catfish *Clarias batrachus*, all of which can be found across most of peninsular Florida (Table 1; Figure 1). Established species which have the most restricted ranges include the eastern happy *Astatotilapia calliptera* and the croaking gourami *Trichopsis vittata*, both of which maintain small, localized populations (Table 1).

Potential ranges of established species based on temperature tolerance varied widely. The species with the smallest potential range based on a lower lethal temperature of 15 degrees Celsius was the butterfly peacock bass *Cichla ocellaris*, which has a potential range of approximately 32,026 km^2^. In contrast, species able to tolerate lower temperatures below 8.6 degrees Celsius can potentially establish populations anywhere in peninsular Florida for a potential range of approximately 141,300 km^2^ (Table 1; Figure 2). Species with this potential ability include Oriental weather loach *Misgurnus anguillicaudatus*, green swordtail *Xiphophorus hellerii*, southern platyfish *Xiphophorus maculatus*, variable platyfish *Xiphophorus variatus*, swamp eel *Monopterus albus/javenensis*, Texas cichlid, blue tilapia, Nile tilapia, Jack Dempsey *Rocio octofasciata*, and croaking gourami (Table 1). Despite their tolerance for cold temperatures, only two of those ten species are considered fast spreaders.

**Figure 3:**
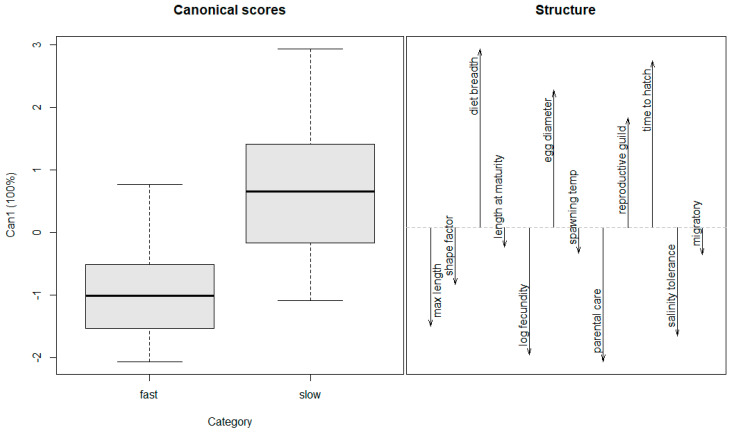
Canonical discriminant function analysis of life history traits. Slow or fast labels in the first pane (Category) are based on the chosen cutoff value of 0.16 (see text). Directionality of variables in the second pane show the combination of traits that best differentiate fast-spreading species from slow-spreading species.

The logistic regression model with the lowest AIC for predicting spread contained three variables including maximum length, length at maturity, and diet breadth (Table 3). The Mann–Whitney U test identified diet breadth (*p* < 0.05) and maximum length (*p* < 0.05) as the only significant variables when comparing fast spreaders to slow spreaders, while length at maturation was nearly significant (*p* = 0.08; Table 4). A logistic regression model with these three terms, and a model with these three terms plus interactions yielded a lower AIC than the final model from the bi-directional stepwise selection process (Table 3). Canonical discriminant analysis suggests that fast spreaders tend to have a larger body size, narrow diet, shorter time to hatch, greater salinity tolerance, and higher fecundity (Figure 3). The reproductive guild trait was correlated with slow spreaders because all four poecilid species are livebearers and were classified as slow spreaders. All established species included in the analyses, except for the Oriental weather loach, have some level of parental care.

## 4. Discussion

The variables that were included in the models separate slow spreaders from fast spreaders but differ almost completely from those that predict reproduction and establishment of non-native freshwater fishes in peninsular Florida [35]. The variables that were included in all models for predicting spread were maximum length and length at maturity. Diet breadth was also highly predictive with slow spreaders exhibiting a more diverse diet. In contrast, the variables that were included in all or most models predicting reproduction were parental care and egg diameter, and those included in most establishment models were parental care, lack of fluvial dependence, and vertical position in the water column, i.e., [35]. Nearly all established non-native fish freshwater species in peninsular Florida have higher levels of parental care, and larger egg diameters [34,35] so there is little use for those variables as a predictor of success beyond the establishment stage. Similarly, none of those established species are fluvial dependent, and all can reproduce and thrive in areas without flowing water.

Predicting whether an established species will spread quickly or slowly in Florida may be more complicated than predicting whether an introduced fish species will establish or fail. With a few exceptions, fast spreaders seem to have a larger body size, narrower diet breadth, higher fecundity and smaller eggs, and salinity tolerance. Many of the faster-spreading species, such as those in the genus *Pterygoplichthys*, are particularly popular in the pet trade and likely have high propagule pressure [39]. Others, such as the blue tilapia, are important food fish and have spread throughout the state both naturally and through assistance by humans [40,41]. It is important to understand characteristics associated with fast spreaders, as newly detected non-native species can be screened for Early Detection Rapid Response needs before populations establish and spread, making containment and eradication no longer feasible.

Another important variable that plays a role in a species’ ability to spread is its climate match/suitability to the assessment area and thermal tolerance [9,42]. Although all fishes considered in this study have a suitable climate in at least portions of peninsular Florida, some species such as the butterfly peacock bass have a very limited cold tolerance [30]. This prevents them from establishing populations north of the Everglades region where water bodies drop below their lower lethal temperature of 15 degrees C during the winter [28]. Other species, such as the brown hoplo have a lower lethal around 10 degrees C which allows them to survive in and spread throughout most of peninsular Florida. Identifying these limitations is relatively simple for areas where USGS water temperature data are available if data are also available on species’ cold tolerance; e.g., [28]. We used lower lethal temperature to determine the potential area that each species could occupy rather than climate match because simple measures of climate similarity can underestimate or overestimate potential range [31].

The mechanisms by which a fish can disperse are numerous and spread may be highly correlated with the strength of the introduction pathways, assuming the receiving environment is suitable for survival [43]. The strongest pathway of initial introduction in Florida is thought to be aquarium release [27]; however, secondary spread of small populations can result either naturally, or from a different set of pathways [44]. Secondary spread can occur naturally, such as dispersal throughout a river system (e.g., silver carp *Hypophthalmichthys molitrix* in the Mississippi River Basin), or between water bodies when moved by humans either intentionally or as a hitchhiker [44]. Florida’s non-native fishes have dispersed through a wide variety of mechanisms, necessitating a more flexible approach for quantifying spread than the methods outlined in previous studies. Some species, such as the goldline snakehead *Channa aurolineata* and the butterfly peacock bass, have both spread throughout the water bodies they can access without assistance and been moved by anglers [45]. Other species maintain relatively disjunct populations and the appearance of spread may be due to repeated introductions (e.g., Jack Dempsey), whereas many widespread species (e.g., blue tilapia) have dispersed quickly via a combination of movement by humans and natural spread within waterbodies [41,46]. Interestingly, several non-native species in Florida are amphibious, breathing air and moving overland unassisted (e.g., walking catfish [47]; and swamp eel [48]). Barriers can also slow or prevent the spread of fishes, including dams and water control structures, features of many otherwise interconnected aquatic systems in Florida. The vast number of highly diverse water bodies in Florida, and limited fish collection data for many areas, also complicates the use of previously established methods for quantifying non-native fish spread [11,12,13,14,22,23,24].

The method used in this study can conservatively estimate spread that may occur through a variety of mechanisms. Florida’s landscape is a mosaic of water bodies [49] which makes counting the number of occupied water bodies infeasible. Many species that are established in Florida are widespread across south Florida, which is composed of only two HUC 8 watersheds. Therefore, if spread was measured by the number of watersheds occupied, species occupying a small area of central Florida may appear to have spread faster than species occupying all of south Florida. To avoid these biases and to account for all mechanisms of spread, we measured the geographic area in which a species appears to have numerous populations that are within close proximity. Other approaches for quantifying spread may be effective for Florida, particularly for analyses of certain species or smaller regions where the number of water bodies occupied is more straightforward.

The non-native fish fauna in previous studies is largely different from those of Florida’s peninsula [34,35] and therefore spread rates, mechanisms of spread, and traits contributing to spread likely vary among study regions. For example, quickly spreading non-native fishes in the Great Lakes had slower growth rates and poorer survival in higher temperatures but could tolerate a wider range of temperatures when compared to slowly spreading fishes [11]. In an analysis of non-native fish spread in California, Marchetti et al. [13] found that more widespread species tend to be long-lived, and that herbivorous fishes tended to be slow spreaders. For non-native fishes in the Iberian Peninsula, Ribeiro et al. [14] identified fast-spreading fishes as those generally having larger body sizes, low levels of parental care, small native range, prior invasion success, and a detritivorous feeding strategy. For non-native fishes in Florida, established species that spread quickly have few traits in common with fast spreaders from the previously mentioned studies. One variable that has been used as a predictor of spread by non-native fishes in California, the Iberian Peninsula, and the present study is feeding strategy. The specific feeding strategy leading to faster spread differs across regions, however.

There are many other factors that may be effective predictors of spread including other life history traits such as growth rate, that were not included due to a lack of available data. This analysis focused primarily on life history characteristics and therefore did not include some commonly used variables such as history of invasion, climate match, and propagule pressure [31]. The inclusion of traits such as these may be useful when assessing risk of invasion, as they have been effective predictors for other regions [11,13,14,50]. In Florida, propagule pressure is likely high for most of the non-native fish species that have established self-sustaining populations due to their popularity as aquarium or food fish [27]. Therefore, the utility of using propagule pressure as a predictor of spread may be limited. Climate match does not seem to be an effective predictor of success for non-native fishes in Florida, as many of the introduced species are from similar donor regions which have produced both successful and unsuccessful species [31]. More complicated interactions such as biotic resistance by native species likely also play a role in limiting spread by some non-native fish species, particularly those that are small-bodied [51,52,53]. Many of the same limitations apply for predicting spread as were discussed in Lawson et al. [35]. These include lack of life history data for many species and a lack of studies examining traits that lead to regional invasion success by non-native fishes.

In conjunction with the results from Lawson and Hill [34,35], the results of this study can be directly applied toward the development of a risk screening tool for peninsular Florida. Further, the methods and results of this study may be useful for regions with a similar hydrology to Florida. Regions with a subtropical climate and little topographical variation may also benefit from these approaches. The traits discussed in the present study may be useful for predicting whether an established species will maintain small, disjunct populations like the green swordtail or Jack Dempsey, or rapidly spread across the study region like the brown hoplo. New research supports a small group of traits, such as high parental care and broad diet, that are becoming highly predictive of establishment success [34,35,54,55,56]. As fewer researchers examine factors that may predict or facilitate fast versus slow spread rates, this work may help grow the body of research that is aimed at better understanding traits that lead to invasion potential rather than just establishment potential [57].

The final stage that needs attention in a stage-based risk screening tool for Florida is the impacts stage. Very little is known about the impacts of most non-native fish species [58], including Florida [29]. To work around this knowledge gap, some argue for the use of the precautionary principle while others equate a lack of information regarding impacts to “no impacts” [58]. There are very few documented effects of non-native fish species in Florida; however, evidence of impacts for several species, particularly *Pterygoplichthys* sp. and swamp eel, is accumulating; e.g., [59,60,61]. Although some interpret the lack of data to mean a lack of impact, continuing research is needed to support such conclusions [29]. An important next step is to summarize the available information on impacts for non-native fish species that have established in Florida, and to develop a flexible framework for quantifying level of impacts. This will help identify information gaps through quantification, and predicting what factors contribute to higher levels of impact may become possible.

## 5. Conclusions

The results of this study suggest that life history traits including maximum body length, diet breadth, and female length at maturation may be useful for predicting fast- versus slow-spreading non-native fishes in Florida. Species classified as fast spreaders tended to have a larger body size and narrower diet breadth, with many also having higher fecundity, smaller eggs, and salinity tolerance compared with slow-spreading species. Traits that were useful for distinguishing non-native fish that have successfully established in Florida versus those that have not, particularly parental care, were less useful for the prediction of spread as nearly all established species have moderate to high levels of parental care [34,35]. This information is useful for screening newly introduced fishes, or those that may be introduced, to understand their potential risk to Florida.

## Figures and Tables

**Table 1:** Calculations of spread by established, non-native fish species in Florida. LLT indicates the lower lethal temperature of each species in degrees Celsius. Spread rate indicates the area colonized per year (km^2^) since introduction, potential range indicates the extension of favorable habitats based on its lower lethal temperature, and percent range per year is the percent of the species’ potential range that it has colonized per year since introduction. Slow or fast labels in the last column (Category) are based on the chosen cutoff value of 0.16 (see text).

Species	Years	Area (km^2^)	Spread Rate	LLT (°C)	Potential Range (km^2^)	% Range Per Yr	Category
*Chitala ornata*	30	605	20	12	111,523	0.018	slow
*Misgurnus anguillicaudatus*	36	7703	214	0	141,300	0.151	slow
*Hoplosternum littorale*	29	80,312	2769	10	128,371	2.157	fast
*Hypostomus* sp.	66	350	5	11.2	111,523	0.005	slow
*Pterygoplichthys* sp.	33	78,025	2364	10	128,371	1.842	fast
*Clarias batrachus*	57	79,130	1388	9.8	128,371	1.081	fast
*Belonesox belizanus*	67	12,221	182	9.7	128,371	0.142	slow
*Xiphophorus hellerii*	54	2439	45	7.5	141,300	0.032	slow
*Xiphophorus maculatus*	54	89	2	7.3	141,300	0.001	slow
*Xiphophorus variatus*	54	210	4	7.3	141,300	0.003	slow
*Monopterus albus/javanensis*	27	9580	355	8	141,300	0.251	fast
*Macrognathus siamensis*	22	2970	135	9	128,371	0.105	slow
*Amphilophus citrinellus*	43	847	20	12	111,523	0.018	slow
*Astronotus ocellatus*	64	21,774	340	12.9	75,038	0.453	fast
*Cichla ocellaris*	40	12,820	321	15	32,026	1.001	fast
*Cichlasoma bimaculatum*	64	30,166	471	8.9	128,371	0.367	fast
*Trichromis salvini*	44	1115	25	9.5	128,371	0.020	slow
*Mayaheros urophthalmus*	40	51,768	1294	10	128,371	1.008	fast
*Astatotilapia calliptera*	26	43	2	10.5	128,371	0.001	slow
*Rubricatochromis letourneuxi*	65	41,136	633	9.5	128,371	0.493	fast
*Herichthys cyanoguttatus*	84	1253	15	5	141,300	0.011	slow
*Heros severus*	31	1420	46	12.8	75,038	0.061	slow
^1^ *Oreochromis aureus*	63	83,005	1318	6.2	141,300	0.932	fast
*Oreochromis mossambicus*	54	3845	71	9.5	128,371	0.055	slow
^1^ *Oreochromis niloticus*	18	22,761	1265	8.2	141,300	0.895	fast
*Parachromis managuensis*	32	7927	248	12	111,523	0.222	fast
*Rocio octofasciata*	64	871	14	8	141,300	0.010	slow
*Sarotherodon melanotheron*	64	3811	60	10.3	128,371	0.046	slow
*Pelmatolapia mariae*	50	36,390	728	11.2	111,523	0.653	fast
*Channa aurolineata*	24	2129	89	10	128,371	0.069	slow
*Trichopsis vittata*	46	3	0	7.2	141,300	0.000	slow

^1^ The occurrence of putative hybrid tilapia with traits of blue tilapia *Oreochromis aureus* and Nile tilapia *Oreochromis niloticus* in Florida dates back to at least 1972 (Hill 2017); morphologically “good” Nile tilapia specimens were noticed in Florida beginning in 2006 (Hill 2017; USGS 2024). Confusion over tilapia identification in Florida may influence the calculated spread rate.

**Table 2:** Life history traits used in the analyses with the range of values across the 31 species as well as the mean and standard deviation of those values.

Trait	Units and Measurement	Range	Mean + SD
Maximum length	Maximum body total length (TL), measured in cm	6.0–183.0	41.8 ± 35.5
Shape factor	Ratio of TL to maximum body depth	2.5–18.5	4.5 ± 3.0
Swim factor	Ratio of minimum depth caudal peduncle to maximum depth caudal fin	0.3–1.5	0.6 ± 0.2
Diet breadth	Range 1–7 (algae, macrophytes, detritus, zooplankton, aquatic insects, macroinverts, fish)	1.0–5.0	2.7 ± 1.1
Length at maturation	Female: measured in cm	2.3–80.0	16.5 ± 17.0
Fecundity	Average number eggs or offspring per breeding season; log10 scale	1.5–4.9	3.1 ± 0.8
Egg size	Mean diameter of mature oocytes in mm	0.8–9.0	2.7 ± 2.2
Spawning temperature	Temperature at which spawning is initiated (Celsius)	20.0–30.0	24.8 ± 2.8
Parental care	Energetic contribution; calculated following Winemiller 1989	0.0–6.0	4.6 ± 1.4
Reproductive guild	Nonguarder, guarder, or livebearer	0.0–2.0	1.1 ± 0.4
Time to hatch	Mean time to egg hatch post-spawn (hours)	24.0–1008.0	173.4 ± 232.8
Salinity tolerance	None (0–4 ppt), low (5–10 ppt), moderate (10–20 ppt), high (>20 ppt)	0.0–3.0	1.7 ± 1.1
Length spawning season	Number of months	2.0–12.0	6.8 ± 3.2
Migratory	Yes (1), no (0)	0.0–1.0	0.4 ± 0.5
Air Breathing	Yes (1), no (0)	0.0–1.0	0.3 ± 0.5

**Table 3:** Logistic regression models with the lowest AIC using bi-directional stepwise selection and inference from paired tests.

Model	AIC
spread group ~ max length × diet breadth × length at maturity	36.51
spread group ~ max length + diet breadth + length at maturity	42.66
spread group ~ max length + length at maturity + fecundity + parental care + salinity tolerance	42.76

**Table 4:** Mann–Whitney U test results for each variable in a comparison of fast versus slow spreading species. Significant values (*p* < 0.05) are denoted with an asterisk.

Species Characteristic	U Statistic	*p*-Value
Maximum body length	180.5	0.012 *
Shape factor	110	0.795
Swim factor	91.5	0.317
Diet breadth	66	0.036 *
Length at maturation	161.5	0.078
Fecundity	145.5	0.262
Egg size	102	0.561
Spawning temperature	125	0.762
Parental care	145.5	0.199
Reproductive guild	97.5	0.234
Time to hatch	108	0.732
Salinity tolerance	144.5	0.260
Length spawning season	115	0.950
Migratory	123	0.791
Air breathing	114	0.902

## Data Availability

The life history trait data for all species included in this study are available on Dryad at the following link: https://doi.org/10.5061/dryad.3n5tb2rjt.
